# Editorial: Integrated role of nutrition and digestive physiology for animal health, Volume II

**DOI:** 10.3389/fvets.2022.1109703

**Published:** 2022-12-28

**Authors:** Demin Cai, Hao-Yu Liu, Haibo Dong, Abdelkareem A. Ahmed

**Affiliations:** ^1^Laboratory of Animal Physiology and Molecular Nutrition, College of Animal Science and Technology, Yangzhou University, Yangzhou, China; ^2^Center for Translational Biomedical Research, University of North Carolina at Greensboro, Kannapolis, NC, United States; ^3^Department of Animal and Veterinary Sciences, Botswana University of Agriculture and Natural Resources, Gaborone, Botswana; ^4^Biomedical Research Institute, Darfur University College, Nyala, Sudan; ^5^Department of Physiology and Biochemistry, Faculty of Veterinary Sciences, University of Nyala, Nyala, Sudan

**Keywords:** integrated nutrition, digestive physiology, host-microbe interaction, diet integration, macronutrients

## Introduction

Integrating nutrition and digestive physiology is crucial for animal health and reproductive performance. The gastrointestinal (G.I.) tract plays a pivotal role in animal health in mammalian species. To perform its maximum function, the digestive system is structured into highly region-specific functional compartments, particularly the histological features of G.I. The histological and functional characteristics of the G.I. commonly reflect the nutritional adaptation of animals. It has been documented that the nutritional types of animals can impact the functions of the G.I., for instance, the intestine and digestive accessory organs such as the liver. Not only the amount of the diet is important, but the characteristics and the quality of the diet are vital in influencing the outcome of animal metabolic health. To illustrate the landscape of the interaction between physiology digestion and nutrition, an integrated perspective is essential, with the application of precise methods and deep-rooted evaluation. Our current Research Topic aims to assemble research of high scientific quality that focuses on incorporating diets and digestive physiology on outcomes related to animal metabolic health. Specifically, the latest advances in the field of digestive physiology to define unique macronutrients, their functions, and signaling pathways at the molecular level will eventually establish the application of new methods, such as omics in animal nutrition and their digestive system. In addition, the Research Topic aimed to collect the recent studies that will contribute to developing new dietary strategies for maintaining digestive system health that is both ecological and economical, and finally to compare and transfer knowledge between experimental animals, livestock, and human being, with the overall aim to improve host fitness.

In this Research Topic, Bai et al. reported the average daily gain and energy and nitrogen requirements of 4-month-old female yak calves. They concluded that the energy and N requirements for yak calves were relatively low. This could be explained, at least in part, by the high efficiency of utilization of energy and high B.V. of N when compared to other livestock. The importance of this study is the benefits of designing early weaning systems for the many Himalayan households depending on yak production for their livelihoods. In addition, Buyse et al. investigated the chestnut tannins in broiler diets: affecting intestinal development in different feeding phases. High doses of various tannins could impair broiler growth, which seems to be linked to lowered protein availability. However, effects on protein digestion under the influence of hydrolyzable tannins were minimal in previous research and literature. Other possible proposed reasons to explain reduced growth are scarce. The authors concluded that tannins may have influenced intestinal growth, both macroscopically as well as histologically. The study hypothesizes that the observed growth reduction with tannins could result from changed energy and nutrient partitioning, i.e., more nutrients are directed to intestinal growth than muscle growth. Antibiotics are widely used in livestock production as growth promoters (AGPs) to improve animal performance and health. However, pig producers face the prohibition of in-feed antimicrobials and must find safe and effective alternatives. *Lactobacillus* species are active microorganisms that convey multiple beneficial effects to the host and are one of the most promising AGPs replacements. Therefore, Zhu et al. summarized the review paper on a meta-analysis of *Lactobacillus*-based probiotics for growth performance and intestinal morphology in piglets. They reported concrete evidence of the growth-promoting effects of *Lactobacillus* spp. and supplementation in piglets and a better understanding of the potential of *Lactobacillus*-based probiotics as AGPs alternatives in pig production. Furthermore, Wang et al. investigated the impacts of tannic acid (T.A.) supplementation at different levels on the growth performance, physiological, oxidative, immunological metrics, and ruminal microflora of Xiangdong black goats. The study concluded that T.A. supplementation decreased C.P. digestion and caused oxidative stress and inflammatory response without influencing growth performance and ruminal microbiota diversity. Collectively, the Research Topic generated and assembled new information in the field of integrated role of nutrition and digestive physiology that will improve animal health.

## Conclusions

The digestive system is the system in physiology with a variety of compartments and functions that play a great complex role in animal health. The typical function of the digestive system is the degradation of feed into its building blocks through enzymes and microflora fermentation, that finally absorbed into the bloodstream. The present Research Topics summarized the research articles that range from the effects of specific nutrients on digestive system physiology, energy balance requirements, unique physiological effects, and some microbial-originated molecules of the digestive system that expand our knowledge. Future research should focus on dietary regimens designed for the prevention of metabolic diseases to improve animal health and the latest advances. In addition to the dietary programs designed as antibiotic alternatives, motivations for and prevalence of use of nutritional supplements in the animal industry.

## Author contributions

AA wrote the introduction and the conclusion. DC wrote the central part with comments on the cited papers and references. H-YL and HD contributed to the review and editing. All authors contributed to the article and approved the submitted version.

